# Human genetics and real‐world patient data fuel target and drug discovery in Alzheimer's disease using Artificial Intelligence

**DOI:** 10.1002/alz70855_101947

**Published:** 2025-12-23

**Authors:** Feixiong Cheng, Yuan Hou, Andrew A. Pieper, Jonathan L Haines, Jeffrey L. Cummings

**Affiliations:** ^1^ Cleveland Clinic, Cleveland, OH, USA; ^2^ Louis Stokes VA Medical Center, Cleveland, OH, USA; ^3^ Case Western Reserve University, Cleveland, OH, USA; ^4^ Louis Stokes Cleveland VA Medical Center, Cleveland, OH, USA; ^5^ Brain Health Medicines Center, Harrington Discovery Institute, University Hospitals Cleveland Medical Center, Cleveland, OH, USA; ^6^ Department of Population and Quantitative Health Sciences, Case Western Reserve University School of Medicine, Cleveland, OH, USA; ^7^ Department of Epidemiology and Biostatistics, Case Western Reserve University, Cleveland, OH, USA; ^8^ Case Western Reserve University School of Medicine, Cleveland, OH, USA; ^9^ Cleveland Institute for Computational Biology, Case Western Reserve University, Cleveland, OH, USA; ^10^ Case Western Reserve University School of Medicine, Department of Population & Quantitative Health Sciences, Cleveland Institute for Computational Biology, Cleveland, OH, USA; ^11^ Departments of Medicine (Biomedical Genetics), Neurology, Ophthalmology, Biostatistics, and Epidemiology, Boston University Schools of Medicine and Public Health, Boston, MA, USA; ^12^ Department of Population and Quantitative Health Sciences, Institute for Computational Biology, Case Western Reserve University, Cleveland, OH, USA; ^13^ Cleveland Institute for Computational Biology, Department of Population & Quantitative Health Sciences, School of Medicine, Case Western Reserve University, Cleveland, OH, USA; ^14^ Chambers‐Grundy Center for Transformative Neuroscience, Department of Brain Health, School of Integrated Health Sciences, University of Nevada Las Vegas, Las Vegas, NV, USA; ^15^ University of Nevada, Las Vegas, Las Vegas, NV, USA; ^16^ Kirk Kerkorian School of Medicine, University of Nevada Las Vegas, Las Vegas, NV, USA; ^17^ Cleveland Clinic Lou Ruvo Center for Brain Health, Las Vegas, NV, USA; ^18^ Chambers‐Grundy Center for Transformative Neuroscience, Las Vegas, NV, USA

## Abstract

**Background:**

Although high‐throughput DNA/RNA sequencing technologies from the NIA‐funded Alzheimer's disease sequencing project (ADSP) have generated massive genetic and genomic data in human disease, translation of these findings into new patient treatment has not materialized by lack of effective approaches, such as Artificial Intelligence (AL) and Machine Learning (ML) tools.

**Method:**

To address this problem, we have developed and utilized AI/ML approaches, Mendelian randomization (MR), and large patient's genetic and functional genomic data to evaluate druggable targets using Alzheimer's disease (AD) as a prototypical example. We utilized the genomic instruments from 9 expression quantitative trait loci (eQTL) and 3 protein quantitative trait loci (pQTL) datasets across five human brain regions from three biobanks. We tested the outcome of Mendelian randomization across genome‐wide association studies (GWAS) datasets with 275,540 AD cases and 1.55 million controls. We searched repurposable drugs using AI‐assistant drugome‐wide association studies from ∼80 million electronic health records.

**Result:**

We identified ∼30 druggable genes supported by significant MR evidences. We pinpointed that an anti‐inflammatory AD target of epoxide hydrolase 2 (EPHX2): (1) a pQTL lead SNP rs2741342 (P_GWAS_ = 5.72×10^‐13^; P_pQTL_ = 1.19×10^‐16^) located in an enhancer of *EPHX2*; and (2) a protein‐coding variant of rs751141 (p.Arg287Gln) was associated with reduced EPHX2 protein expression (P_pQTL_ = 5.50×10^‐16^) from the AD knowledge portal. We experimentally validated that the EPHX2‐p.Arg287Gln (a protective mutation) significantly reduced the level of phosphorylated‐tau (ptau181) and increased neuron clumped size in AD patient iPSC‐derived neurons. We demonstrated that EC5026 (a first‐in‐class, picomolar EPHX2 inhibitor) improves cognition in a 5xFAD mouse model. Wefurther identified that 12 drugs (i.e., trazodone [ADRA1A] and baclofen [GABBR1]) harboring MR‐supported targets are significantly associated with reduced incidence of AD in 111,680 mild cognitive impairment (MCI) patients.

**Conclusion:**

Combining genetics and real‐world patient data via AI/ML technologies identifies potential therapeutic targets and medicines for AD. Further functional and clinical validation of candidate targets and drugs in ethnically diverse population are warranted.

